# Cancer incidence among seafarers and fishermen in the Nordic countries

**DOI:** 10.5271/sjweh.3879

**Published:** 2020-09-01

**Authors:** Kajsa Ugelvig Petersen, Eero Pukkala, Jan Ivar Martinsen, Elsebeth Lynge, Laufey Tryggvadottir, Elisabete Weiderpass, Kristina Kjærheim, Sanna Heikkinen, Johnni Hansen

**Affiliations:** 1 Danish Cancer Society Research Center, Danish Cancer Society, Copenhagen, Denmark; 2 Finnish Cancer Registry, Institute for Statistical and Epidemiological Cancer Research, Helsinki, Finland; 3 Faculty of Social Sciences, Tampere University, Tampere, Finland; 4 Department of Research, Cancer Registry of Norway, Oslo, Norway; 5 Nykøbing Falster Hospital, University of Copenhagen, Copenhagen, Denmark; 6 Icelandic Cancer Registry, Reykjavik, Iceland; 7 Faculty of Medicine, BMC, Laeknagardur, University of Iceland, Reykjavik, Iceland; 8 International Agency for Research on Cancer, Lyon, France

**Keywords:** carcinogenic chemical, maritime health, mariner, maritime worker, NOCCA, Nordic Occupational Cancer Study, occupational exposure, sailor, seaman, ultraviolet radiation, working environment

## Abstract

**Objectives:**

Maritime workers may be exposed to several occupational hazards at sea. The aim of this study was to assess cancer incidence among seafarers and fishermen in the Nordic countries and identify patterns in morbidity in the context of existing studies in this field.

**Methods:**

A cohort of 81 740 male seafarers and 66 926 male fishermen was established from census data on 15 million citizens in the five Nordic countries. Using personal identity codes, information on vital status and cancer was linked to members of the cohort from the national population and cancer registries for the follow-up period 1961–2005. Standardized incidence ratios (SIR) were calculated applying national cancer incidence rates for each country and pooling results.

**Results:**

The overall incidence of cancer was increased among the male seafarers [SIR 1.22, 95% confidence interval (CI) 1.19–1.23]. Significant excesses were observed for multiple cancer sites among the seafarers, while results for the fishermen were mixed. Lip cancer incidence was increased among both maritime populations. For mesothelioma (SIR 2.17, 95% CI 1.83–2.56 seafarers) and non-melanoma skin cancer (SIR 1.23, 95% CI 1.14–1.32 seafarers), incidence was increased among the seafarers.

**Conclusion:**

In our cohort, seafaring was associated with a higher overall incidence of cancer compared to the general population. While the majority of cancers could not be linked to specific occupational factors, increases in mesothelioma, lip and non-melanoma-skin cancer indicate previous exposure to asbestos, ultraviolet radiation and potentially also chemicals with dermal carcinogenic properties at sea.

Spending most of their professional life at sea, merchant seafarers and fishermen may share several occupational hazards. Both maritime populations risk exposure to a range of carcinogenic chemicals released by ship engines through material wear or used in production or maintenance work on board (ie, diesel exhaust, asbestos and polycyclic aromatic hydrocarbons) (1–3). With long hours on duty and frequent night-shifts, seafarers and fishermen may also suffer potential disruption of circadian rhythms and fatigue. Finally, ultraviolet (UV) radiation can be more intense at sea with the added reflection from water and ship surfaces and travel at near-equatorial latitudes or in areas with ice and snow (1).

Despite these expected similarities in working conditions, several other important factors may limit comparability in health between seafarers and fishermen. While seafaring traditionally has attracted a range of personalities suited for isolation and adventure, fishermen have often been recruited from within maritime communities and families (4–6). Many seafarers spend long periods at sea separated from their social and family life and their resulting lifestyle may, thus, differ from the norm among fishermen, who return home regularly (5).

Maritime workers have a history of problems with obesity due especially to limited awareness of and options for diet and exercise on board (1, 7–10). While fishermen tend to have a high intake of fish and still face demanding physical workloads, technical advances and automation have widely reduced previous demands for muscle power among seafarers (5, 10–13). In turn, seafarers risk exposure to potentially toxic cargoes, especially on chemical and product tankers (5, 14–16).

In previous studies, the risk of cancer among seafarers and fishermen has differed from that observed in corresponding general populations. Several studies have shown potentially work-related increases in the incidence of lip cancer among both seafarers and fishermen (12, 13, 17,18). While studies have also indicated an occupational association with mesothelioma for seafarers, no evidence of an excess risk for this specific cancer has been found among fishermen (17, 19–21). The aim of our present study was to assess cancer patterns in the Nordic countries with a focus on work-relation in a large pooled cohort of maritime workers employed over several decades in the context of the existing knowledge in this particular field. Based on previous findings, cancer of the lip and mesothelioma were outcomes of a priori interest (12, 13, 17–19).

## Methods

The process of data collection in the Nordic Occupational Cancer (NOCCA) project and analyses involved have been described in detail previously (22). In brief, the subjects included were participants from a number of population censuses in Denmark (1970), Finland (1970, 1980 and 1990), Iceland (1981), Norway (1960, 1970 and 1980), and Sweden (1960, 1970, 1980 and 1990). The remaining criteria for inclusion in our cohort were residency in the country of census on 1 January of the year following the census with an age of 30–64 years and a self-reported occupation in the field of either seafaring or fishing. Census questionnaires contributed freetext information on personal economy, education, occupation, industry of employment and employer (22, 23). Questionnaire data were initially coded nationally and subsequently converted to a common occupational coding system with seafarers and fishermen listed as two separate groups (22). The group of seafarers consisted of officers, machinists, deck and engine room crew, ship maintenance, and repair workers. In addition to actual fishermen, the latter group also included specialized whale and seal hunters (only present in Iceland and Norway) (24).

In all five Nordic countries, subjects included were uniquely identifiable through the use of personal identity codes (22). These codes have served as a personal key for all permanent residents and tax payers in all public administration in the entire study period (25). Using personal identity codes, information on death and emigration was linked to each subject in the cohort from the respective Population Registry in each country (22).

### Cancer

All cancers diagnosed in the Nordic countries are registered systematically in nationwide population-based cancer registries (26, 27). As these registries receive notifications on specific diagnoses of cancer from practitioners, hospitals, institutions, laboratories, and other health registers, the information held is considered highly valid and complete (26, 27). Though sources and coding procedures have varied slightly between the different countries and time periods included, continuous efforts to standardize the data collected on cancer has ensured a high level of comparability across the Nordic region (22, 26, 27).

In our study, information on cancer was linked through personal identity codes to members of the cohort from the cancer registry in the country of census. Follow-up began on 1 January of the year following first census participation (Denmark 1971, Finland 1971, 1981 and 1991, Iceland 1982, Norway 1961, 1971 and 1981, and Sweden 1961, 1971, 1981 and 1991) and ended on the first of either date of death, emigration or 31 December in the final study year for each country (Denmark and Norway 2003, Iceland 2004, Finland and Sweden 2005) (22). The subsequent classification of cancer was based on 49 main groups with 27 subcategories combining detailed information on both topography and morphology (24). All primary malignancies and benign brain tumors diagnosed during follow-up were counted in our analyses for cancer incidence. Thus, a person experiencing more than one primary cancer in the course of follow-up would contribute several diagnoses. However, in the data from Denmark, Iceland and Sweden, only the first primary cancer in each of the 49 groups was included (22). While this approach caused a slight dissymmetry in the absolute numbers of cancers observed in the respective countries, it had no actual effect on the precision of the resulting standardized incidence ratios (SIR) (23).

### Statistical analysis

For each member of the cohort, person-years at risk were calculated for the relevant period of follow-up and split into 5-year age and calendar time intervals. Using the national incidence rates for the same intervals and approach counting all primary cancers for each of the five Nordic countries, the expected numbers of overall and site-specific cancers were then calculated for seafarers and fishermen separately. Dividing the number of observed cases in the cohort by the expected number, SIR were presented for each occupation in each country. Finally, an overall SIR for all countries combined was estimated as the sum of all observed versus the sum of all expected national cases. Assuming a Poisson distribution for the observed cases, 95% confidence intervals (CI) were calculated for each SIR.

In our analyses, non-melanoma skin cancer was excluded from the overall cancer estimate based on a lack of morphological subtype distinction for this specific outcome in the Danish data. Analyses stratifying results according to time period (1961–1975, 1976–1990, 1991–2005) of and age (30–49, 50–69, +70 years) at follow-up were performed subsequently. Only results for the male seafarers and fishermen are presented in the following sections, as the number of census participating women in these professions was insufficient for meaningful interpretation of associations (N=387 seafarers and N=1383 fishermen) (22). As results according to age at follow-up contributed no substantial, additional information, these are limited to the supplementary material (www.sjweh.fi/show_abstract.php?abstract_id=3879).

### Results

The final cohort consisted of 81 740 male seafarers and 66 926 male fishermen. During follow-up, the seafarers and fishermen contributed 2 120 656 and 1 751 709 person-years, respectively. In both groups, the majority of participants were included from the Norwegian censuses (seafarers 53% and fishermen 64%). The specific distribution of persons and person-years included from each country is shown in table 1.

**Table 1:** Characteristics of the cohort of male Nordic seafarers and fishermen; 1961–2005.

Area	Seafarers		Fishermen	
	
	N	Person-years	N	Person-years
Total	81 740	2 120 656	66 926	1 751 709
Denmark	8936	209 956	7053	170 809
Finland	10 230	237 934	2970	65 437
Iceland	1001	19 024	4907	94 090
Norway	42 936	1 182 959	42 711	1 170 043
Sweden	18 637	470 783	9285	251 330

Among the male seafarers, a total of 19 228 cancers were diagnosed during follow-up (2243 in Denmark, 1794 in Finland, 145 in Iceland, 10 647 in Norway and 4399 in Sweden). Their overall incidence of cancer was significantly increased based on consistent excesses in all the Nordic countries (SIR 1.22, 95% CI 1.19–1.23 for all cancers excluding non-melanoma skin cancer). In table 2, the pooled Nordic results for cancer outcomes with >20 cases are presented. Significant increases were observed for cancer of the lip, tongue, oral cavity, pharynx, oesophagus, stomach, colon, liver, pancreas, larynx, lung, mesothelium, prostate, kidney, renal pelvis, urinary bladder, bones, and skin (non-melanoma) among the seafarers (table 2). In the analyses stratifying on time period, the excess of mesothelioma was particularly clear in the later periods of follow-up (table 3). The incidence of lymphohematopoietic cancers was generally not increased and, for non-Hodgkin lymphoma, the incidence was reduced significantly (table 2).

**Table 2:** Cancer incidence among 148 666 male Nordic seafarers and fishermen; 1961–2005. Outcomes with >20 observed cases shown. **Statistically significant results marked in bold**. [ICD-7=international classification of diseases revision 7; Obs=observed; SIR=standardized incidence ratio; CI=confidence interval]

ICD-7	Cancer site	Seafarers (N=81 740)			Fishermen (N=66 926)		
	
		Obs	SIR	95% CI	Obs	SIR	95% CI
140–204	All cancers (minus non–melanoma skin)	18 524	**1.22**	**1.19–1.23**	15 856	1.02	1.00–1.04
140	Lip	185	**1.19**	**1.03–1.38**	384	**2.26**	**2.04–2.50**
141	Tongue	108	**1.66**	**1.36–2.00**	40	**0.68**	**0.49–0.93**
142	Salivary glands	30	0.88	0.60–1.26	34	1.06	0.74–1.48
143–144	Oral cavity	191	**2.04**	**1.76–2.35**	84	0.94	0.75–1.16
145–148	Pharynx	241	**2.06**	**1.80–2.33**	93	0.88	0.71–1.08
150	Oesophagus	332	**1.64**	**1.47–1.83**	205	1.01	0.87–1.15
151	Stomach	1075	**1.23**	**1.15–1.30**	1428	**1.36**	**1.29–1.43**
152	Small intestine	69	1.12	0.87–1.42	60	1.04	0.80–1.34
153	Colon	1397	**1.10**	**1.05–1.16**	1271	**0.94**	**0.88–0.99**
154	Rectum, rectosigma	885	1.07	1.00–1.15	791	0.93	0.86–1.00
155	Liver	270	**1.80**	**1.59–2.03**	111	**0.78**	**0.64–0.93**
155.1	Gallbladder	100	1.19	0.97–1.45	93	1.06	0.86–1.30
157	Pancreas	580	**1.16**	**1.07–1.26**	596	**1.10**	**1.01–1.19**
160	Nasal cavity	41	1.03	0.74–1.39	56	1.32	1.00–1.72
161	Larynx	378	**1.83**	**1.65–2.03**	241	**1.19**	**1.05–1.35**
162–163	Lung	3582	**1.62**	**1.57–1.68**	2546	**1.16**	**1.12–1.21**
158,162.2	Mesothelioma	143	**2.17**	**1.83–2.56**	26	**0.44**	**0.29–0.65**
170	Breast	23	0.97	0.61–1.45	25	1.01	0.66–1.50
177	Prostate	3613	**1.05**	**1.02–1.09**	3229	**0.89**	**0.86–0.92**
178	Testis	90	0.85	0.68–1.05	72	0.90	0.70–1.13
179.0	Penis	57	1.10	0.84–1.43	45	0.83	0.61–1.11
180	Kidney	628	**1.16**	**1.07–1.26**	572	1.08	1.00–1.18
180.1	Renal pelvis	105	**1.51**	**1.23–1.82**	58	0.83	0.63–1.07
181	Urinary bladder	1478	**1.22**	**1.16–1.28**	1450	**1.14**	**1.08–1.20**
190	Melanoma	545	1.05	0.97–1.15	216	**0.51**	**0.44–0.58**
191	Non–melanoma skin	704	**1.23**	**1.14–1.32**	469	**0.75**	**0.68–0.82**
192	Eye	44	0.99	0.72–1.33	38	0.90	0.64–1.23
193	Brain	407	0.97	0.88–1.07	327	0.89	0.80–1.00
194	Thyroid	80	1.10	0.87–1.37	93	**1.32**	**1.06–1.62**
	Follicular	2	**0.27**	**0.03–0.99**	5	0.68	0.22–1.58
	Papillary	45	**1.43**	**1.04–1.91**	51	**1.70**	**1.27–2.23**
196	Bone	36	**1.59**	**1.11–2.20**	25	1.20	0.77–1.77
197	Soft tissue	78	1.01	0.80–1.26	52	**0.72**	**0.54–0.95**
200, 202	Non–Hodgkin lymphoma	367	**0.88**	**0.79–0.97**	348	0.97	0.87–1.07
201	Hodgkin lymphoma	69	0.93	0.73–1.18	59	0.87	0.66–1.12
203	Multiple myeloma	217	0.87	0.76–1.00	258	0.96	0.84–1.08
204	Leukaemia	343	0.90	0.80–1.00	312	**0.78**	**0.70–0.87**
199	Ill–defined/unspecified	587	**1.26**	**1.16–1.36**	622	**1.16**	**1.07–1.25**
	Remaining not shown	38	1.15	0.82–1.58	23	0.73	0.46–1.10

**Table 3:** Cancer incidence by era of follow–up among 148 666 male Nordic seafarers and fishermen; 1961–2005. **Statistically significant results marked in bold.** [Obs=observed; SIR=standardized incidence ratio; CI=confidence interval]. Outcomes of a priori interest or with >1000 observed cases shown. When the observed number of cases is zero, the expected number is presented in parentheses.

Cancer site	Era of follow–up					

	1961–1975		1976–1990		1991–2005	
		
	Obs	SIR (95% CI)	Obs	SIR (95% CI)	Obs	SIR (95% CI)
Seafarers						
Lip	48	1.28 (0.94–1.69)	73	1.04 (0.82–1.31)	64	**1.36 (1.04–1.73)**
Stomach	256	**1.18 (1.04–1.34**)	438	**1.18 (1.07–1.30)**	381	**1.32 (1.19–1.45)**
Colon	131	0.97 (0.81–1.15)	499	1.10 (1.00–1.20)	767	**1.13 (1.05–1.21)**
Lung	492	**1.73 (1.58–1.89)**	1464	**1.62 (1.53–1.70)**	1626	**1.59 (1.52–1.67)**
Mesothelioma	4	1.33 (0.36–3.41)	54	**2.34 (1.76–3.06)**	85	**2.14 (1.71–2.65)**
Prostate	276	**1.15 (1.01–1.29)**	1109	1.07 (1.00–1.13)	2228	1.03 (0.99–1.08)
Urinary bladder	162	**1.37 (1.17–1.60)**	554	**1.18 (1.09–1.29)**	762	**1.22 (1.14–1.31)**
Fisherman						
Lip	123	**2.32 (1.93–2.77)**	178	**2.27 (1.95–2.63)**	83	**2.15 (1.71–2.67)**
Stomach	463	**1.37 (1.25–1.50)**	631	**1.40 (1.29–1.51)**	334	**1.29 (1.15–1.43)**
Colon	171	0.87 (0.75–1.01)	492	**0.89 (0.82–0.98)**	608	0.99 (0.92–1.08)
Lung	373	0.95 (0.86–1.05)	1110	**1.14 (1.08–1.21)**	1063	**1.29 (1.21–1.37)**
Mesothelioma	2	0.54 (0.06–1.94)	10	**0.41 (0.20–0.76)**	14	**0.46 (0.25–0.77)**
Prostate	326	**0.79 (0.70–0.88)**	1281	**0.92 (0.87–0.97)**	1622	**0.89 (0.85–0.93)**
Urinary bladder	161	0.97 (0.83–1.13)	648	1.16 (1.07–1.25)	641	1.16 (1.07–1.25)

Among the male fishermen, the overall number of cancers observed was 16 325 (1821 in Denmark, 538 in Finland, 436 in Iceland, 11 150 in Norway and 2380 in Sweden). In this group, the overall incidence of cancer was on par with that of the general populations in the Nordic countries (SIR 1.02, 95% CI 1.00–1.04 for all cancers minus non-melanoma skin cancer). The pooled results for the individual cancer sites were, however, mixed with several notable increases and decreases in incidence (table 2). Significant excesses were observed for cancer of the lip, stomach, pancreas, larynx, lung, urinary bladder and thyroid. The SIR for leukemia, tongue, colon, liver, prostate, soft tissue, melanoma, and non-melanoma skin cancer were significantly reduced. Contrary to the seafarers, the fishermen also had a significantly decreased incidence of mesothelioma.

Results were relatively consistent across the five included countries (table 4).

**Table 4:** Cancer incidence by country among 148 666 male Nordic seafarers and fishermen; 1961–2005. **Statistically significant results marked in bold**. [Obs=observed; SIR=standardized incidence ratio; CI=confidence interval]. Outcomes of a priori interest or with more than 1000 observed cases shown. When the observed number of cases is zero, the expected number is presented in parentheses.

Cancer site	Seafarers									

	Denmark (N=8936)		Finland (N=10 230)		Iceland (N=1001)		Norway (N=42 936)		Sweden (N=18 637)	
				
	Obs	SIR (95% CI)	Obs	SIR (95% CI)	Obs	SIR (95% CI)	Obs	SIR (95% CI)	Obs	SIR (95% CI)
All cancers ^a^	2243	**1.27 (1.21–1.32**)	1748	**1.20 (1.14–1.25)**	141	**1.26 (1.06–1.48)**	10 231	**1.20 (1.17–1.22**)	4161	**1.24 (1.20–1.27)**
Lip	28	1.44 (0.96–2.09)	23	1.25 (0.79–1.87)	0.62	0	92	1.06 (0.85–1.30)	42	**1.42 (1.03–1.92)**
Stomach	88	**1.32 (1.06–1.63)**	90	1.08 (0.86–1.32)	7	1.16 (0.47–2.39)	678	**1.27 (1.17–1.36**)	212	**1.15 (1.01–1.32)**
Colon	195	**1.26 (1.09–1.46)**	91	1.24 (1.00–1.52)	13	1.50 (0.80–2.56)	813	1.05 (0.98–1.12)	285	1.12 (1.00–1.26)
Lung	546	**1.42 (1.30–1.54)**	353	**1.19 (1.07–1.32)**	23	1.57 (0.99–2.35)	2036	**1.74 (1.67–1.82)**	624	**1.82 (1.68–1.97)**
Mesothelioma	26	**2.51 (1.64–3.68)**	18	**2.68 (1.59–4.23)**	1	2.48 (0.07–15.80)	61	**1.74 (1.33–2.24)**	37	**2.77 (1.95–3.81)**
Prostate	263	1.13 (0.99–1.27)	418	**1.19 (1.09–1.32)**	25	0.84 (0.55–1.25)	1860	0.99 (0.94–1.03)	1047	**1.12 (1.05–1.18)**
Urinary bladder	231	1.19 **(1.04–1.35**)	108	**1.34 (1.11–1.61)**	12	1.42 (0.74–2.48)	813	**1.21 (1.13–1.29)**	314	**1.24 (1.11–1.38)**
	Fishermen									
	
	Denmark (N=7053)		Finland (N=2970)		Iceland (N=4907)		Norway (N=42 711)		Sweden (N=9285)	
					
	Obs	SIR (95% CI)	Obs	SIR (95% CI)	Obs	SIR (95% CI)	Obs	SIR (95% CI)	Obs	SIR (95% CI)
	
All cancers ^a^	1821	**1.12 (1.07–1.17)**	522	0.94 (0.86–1.03)	429	1.07 (0.97–1.18)	10 870	1.02 (1.00–1.04)	2214	0.97 (0.92–1.01)
Lip	57	**3.19 (2.42–4.14)**	14	1.79 (0.98–3.00)	1	0.51 (0.01–2.84)	232	**1.92 (1.69–2.19)**	80	**3.65 (2.90–4.55)**
Stomach	80	1.26 (1.00–1.57)	35	0.99 (0.69–1.37)	25	1.21 (0.78–1.79)	1113	**1.41 (1.33–1.50)**	175	**1.23 (1.06–1.43)**
Colon	154	1.07 (0.91–1.25)	22	0.80 (0.50–1.22)	32	1.06 (0.72–1.49)	892	**0.91 (0.85–0.97)**	171	0.95 (0.82–1.11)
Lung	460	**1.28 (1.17–1.41)**	127	1.02 (0.86–1.22)	78	**1.52 (1.20–1.89)**	1654	**1.17 (1.11–1.22)**	227	0.96 (0.84–1.09)
Mesothelioma	4	0.43 (0.12–1.10)	1	0.49 (0.01–2.71)	1	0.74 (0.02–4.13)	16	**0.43 (0.24–0.69)**	4	0.49 (0.13–1.25)
Prostate	203	0.89 (0.77–1.03)	127	0.97 (0.81–1.15)	95	0.98 (0.80–1.20)	2219	**0.88 (0.84–0.92)**	585	**0.89 (0.82–0.97**)
Urinary bladder	205	1.13 (0.98–1.30)	23	0.71 (0.45–1.07)	34	1.15 (0.80–1.61)	1005	**1.17 (1.10–1.24)**	183	1.05 (0.91–1.22)

a All cancers minus non-melanoma skin cancer.

## Discussion

In this large study on male seafarers and fishermen, we assessed cancer patterns across the Nordic region. The overall incidence of cancer was increased only among the seafarers, while cancer morbidity among the fishermen was on par with that of the respective general populations in the Nordic countries. Among the seafarers, we observed excesses of a wide range of cancer types. The pattern among the fishermen was more mixed with elevated or reduced numbers of observed cases for several specific types of cancer.

With few exceptions, previous studies on cancer in maritime workers support our main findings (12, 13, 17–19, 28). Thus, the overall excess of cancer in seafarers is evident also in a recent Danish study by Petersen et al (17, 29). The large Danish cohort of seafarers included in this study was employed in the years 1986–1999 and, thus, decades after the Danish census in 1970 used in the NOCCA project (17). In contrast, an earlier study by Pukkala et al (19) shows no increase in overall cancer among Finnish seafarers employed in the period 1960–1980. For the fishermen, the non-elevated overall cancer incidence in our cohort is generally corroborated by existing studies (12, 13, 18). A modest overall excess of cancer was observed previously by Rafnsson et al (28) in Iceland, but the cohort examined in that study consisted primarily of fishermen with an undetermined number of seafarers as well. Inconsistencies in results between studies may reflect either a development over time or differences in the actual populations included.

The observed significant increase in lip cancer incidence among both the seafarers and fishermen in our cohort supports existing evidence of an association with this outcome for maritime workers (12, 13, 17,18). Though tobacco usage may play a role in the development of mainly lower lip cancer through especially pipe smoking, occupational exposure to UV radiation likely contributes to this excess risk (30, 31). While specialized offshore clothing protects most of the body during outdoor work at sea, UV radiation can affect uncovered, smaller areas such as the lips intensely (32). As merchant seafarers often travel and work at low latitudes with extreme levels of UV radiation, the excess in non-melanoma skin cancer in this group may also be work-related. Dermal exposure to carcinogens, including soot and polycyclic aromatic hydrocarbons, on board merchant vessels adds to the occupational concerns for this outcome (1, 33, 34). In contrast, the incidence of both melanoma and non-melanoma skin cancer is significantly reduced among the fishermen in our cohort, who are confined to securing their catch in Northern waters.

Our study also confirms the previously established association between occupational seafaring and excess risk of mesothelioma (17, 19). With a profile combining fire and heat resistance, electrical insulation and tensile strength, asbestos containing materials were previously used extensively in all areas of merchant ships with the highest risk of fiber exposure in the engine rooms (3, 17). Due to a very gradual and rather late ban on asbestos on ships, many modern merchant vessels still contain limited amounts of these silicate minerals (35, 36). According to the international conventions for Safety of Life at Sea (SOLAS), asbestos was fully prohibited in new installations from 2011 with the exception of ships build before this point (35). Thus, all the seafarers in our cohort may at some point have been exposed occupationally to asbestos. The latency in development of mesothelioma following exposure to asbestos often spans several decades explaining the notable increase in the later periods of follow-up for this outcome. At the same time, the significantly lower incidence of mesothelioma observed among the fishermen in our study suggests no exposure to asbestos on commercial fishing boats in the Nordic region.

The presence of other airborne chemicals such as diesel exhaust, benzene or polycyclic aromatic hydrocarbons on ships may also have contributed to the increase in lung cancer observed in seafarers and fishermen in our cohort (1, 33). Excretion of especially polycyclic aromatic hydrocarbons has also been linked to both kidney and bladder cancer (34). Similarly, work involving (un)loading or cleaning of (ie, vinyl chloride) chemical tanks may explain some of the excess in liver cancer among the seafarers (5, 33). However, assessing an occupational contribution to these specific cancers is not possible without information on health behavior. Among the seafarers, cancers related to tobacco and/or alcohol usage are almost consistently increased (tongue, oral cavity, pharynx, oesophagus, stomach, colon, pancreas, liver, larynx, lung, kidney, renal pelvis, urinary bladder) (33). For the fishermen, a less pronounced pattern of excess in several tobacco-related cancers also appears (stomach, pancreas, larynx, lung, urinary bladder).

Contrasting the notable excess of many individual cancers among the seafarers, our observation of lower incidence of non-Hodgkin lymphoma in this group has no obvious explanation at this point and may well – with our multiple comparisons – be a random finding. While previous measures have revealed high levels of benzene in both working areas and living quarters on tankers, the incidence of lymphohematopoietic cancers is not increased among the seafarers in our study (16, 17). However, considering the historic composition of the Nordic merchant fleets, the fraction of the seafarers actually employed and exposed on tankers is likely limited in this cohort (37).

Only few studies have reported findings for thyroid cancer among fishermen and none of them confirms the significant excess observed for this outcome in our cohort (12, 28). As thyroid cancer may be associated with obesity, problems with overweight among fishermen could contribute to this result (7, 38). However, the documented high dietary intake of fish among these men may, as a major source of iodine, also play a role in the development of this particular cancer (12). While supplementing dietary iodine seems to reduce the incidence of follicular thyroid cancer in iodine deficient populations, papillary thyroid cancer, on the other hand, is associated with high dietary iodine levels (39, 40). Thus, the specific subtype distribution with low follicular and high papillary thyroid cancer incidence among both the fishermen and seafarers in our cohort supports an association with excessive dietary (fish) intake.

Lifestyle-related factors may also contribute to the lower incidence of several cancers among the fishermen (tongue, colon, liver, prostate, soft tissue cancer and leukemia). With no clear established pattern for these outcomes in previous studies, these results could, however, also represent a healthy worker effect from the comparison of physically active fishermen to general populations or simply random findings (12, 13, 18, 28, 41).

With the limitations of the included census data, no information on either lifestyle or detailed job descriptions or employment durations on our seafarers and fishermen is available. Among seafarers, cancer patterns have varied immensely in previous studies according to both job position and type of ship sailed (17, 20). Similarly, issues with poor health differ among coastal fishermen and those employed on larger, long-distance trawlers (10). Despite potential variations in the types of vessels included in the national merchant and fishing fleets, differences in cancer incidence remain relatively small across the five included countries in our current study (37).

Since the industry recession and changes in crew regulations in the 1980s, Nordic merchant fleets have widely depended on multinational crews to minimize labor costs (1, 5). As foreign seafarers represent other lifestyles, occupy low skill positions, and endure prolonged periods at sea, the pattern of cancer in this group may not resemble that of our Nordic residents completely (1). Finally, the low number of census participating women in these professions also impede the drawing of any conclusions on their part.

As a major strength, our study presents long-term follow-up of a very large cohort of maritime workers. In addition, information on cancer, emigration and death is contributed by high quality registers from the entire Nordic region.

### Concluding remarks

Living and working at sea is associated with specific patterns in cancer morbidity. While the Nordic seafarers in our cohort suffered from a higher overall incidence of cancer compared to the general population, the majority of their cancer cases were not related to specific occupational factors. However, the excess risk observed for mesothelioma and non-melanoma skin cancer among seafarers and lip cancer among both seafarers and fishermen indicates previous exposure to asbestos, intense ultraviolet radiation and potentially also chemical dermal carcinogens at sea.

In recent decades, substantial changes in regulation of health and safety at sea have been implemented. Continued efforts to examine the resulting changes in cancer among maritime workers are therefore recommended.

## Ethical approval

The ethical committees and the data inspection boards in each of the Nordic countries approved the NOCCA study.

## Conflicts of interest

None declared.

## Funding

The NOCCA project was funded by the Nordic Cancer Union.

## Disclaimer

Where authors are identified as personnel of the International Agency for Research on Cancer / World Health Organization, the authors alone are responsible for the views expressed in this article and they do not necessarily represent the decisions, policy or views of the International Agency for Research on Cancer / World Health Organization.

## Supplementary material

Supplementary material
