# Supplementary material for: Cancer incidence among seafarers and fishermen in the Nordic countries
Source: Scand J Work Environ Health. 2020 Sep 1;46(5):461–8. doi: 10.5271/sjweh.3879 (PMC7737810; doi:10.5271/sjweh.3879)
Supplement: Supplementary material [file SJWEH-46-461-S001.pdf]

Cancer incidence among seafarers and fishermen in the Nordic countries [1]

by Kajsa Ugelvig Petersen, PhD, [2] Eero Pukkala, PhD, Jan Ivar Martinsen, Elsebeth Lynge, PhD, Laufey Tryggvadottir, PhD, Elisabete Weiderpass, PhD, Kristina Kjærheim, PhD, Sanna Heikkinen, PhD, Johnni Hansen, PhD

1. *Supplementary material*
2. *Correspondence to: Kajsa Petersen, Danish Cancer Society Research Center, Danish Cancer Society, Strandboulevarden 49, 2100 København Ø, Denmark. [E-mail: kajpet@cancer.dk]*

Table S1 Cancer incidence by age at follow-up among 148 666 male Nordic seafarers and fishermen; 1961-2005.

	**Seafarers**					
	**Age at follow-up**					
	**30-49**		**50-69**		**+ 70**	
**Cancer site**	**Obs**	**SIR (95% CI)**	**Obs**	**SIR (95% CI)**	**Obs**	**SIR (95% CI)**
Lip	21	1.38 (0.85-2.11)	110	**1.26 (1.03-1.51)**	54	1.04 (0.78-1.35)
Stomach	83	**1.31 (1.05-1.63)**	582	**1.27 (1.16-1.37)**	410	**1.16 (1.05-1.28)**
Colon	77	1.18 (0.93-1.47)	679	**1.13 (1.04-1.22)**	641	1.07 (0.99-1.16)
Lung	207	**2.01 (1.74-2.30)**	2157	**1.65 (1.58-1.72)**	1218	**1.52 (1.44-1.61)**
Mesothelioma	10	**3.07 (1.47-5.65)**	67	**1.84 (1.42-2.33)**	66	**2.53 (1.96-3.22)**
Prostate	13	0.87 (0.46-1.49)	1532	**1.07 (1.01-1.12)**	2068	1.04 (0.99-1.09)
Urinary bladder	60	1.17 (0.89-1.51)	737	**1.23 (1.14-1.32)**	681	**1.22 (1.13-1.32)**
	**Fishermen**					
	**Age at follow-up**					
	**30-49**		**50-69**		**+ 70**	
**Cancer site**	**Obs**	**SIR (95% CI)**	**Obs**	**SIR (95% CI)**	**Obs**	**SIR (95% CI)**
Lip	41	**3.90 (2.80-5.28)**	204	**2.31 (2.01-2.65)**	139	**1.95 (1.64-2.30)**
Stomach	90	**1.95 (1.57-2.40)**	703	**1.40 (1.30-1.51)**	635	**1.26 (1.16-1.36)**
Colon	46	1.09 (0.79-1.45)	508	0.94 (0.86-1.03)	717	**0.92 (0.85-0.99)**
Lung	64	0.96 (0.74-1.23)	1371	**1.19 (1.13-1.26)**	1111	**1.14 (1.08-1.21)**
Mesothelioma	(1.61)	0	13	**0.49 (0.26-0.84)**	13	**0.43 (0.23-0.73)**
Prostate	8	0.85 (0.37-1.68)	995	**0.85 (0.80-0.91)**	2226	**0.91 (0.87-0.95)**
Urinary bladder	33	1.00 (0.69-1.41)	633	**1.19 (1.10-1.28)**	784	**1.10 (1.03-1.18)**

Outcomes of a priori interest or with more than 1000 observed cases shown.
When the observed number of cases is zero, the expected number is presented in parentheses.
Statistically significant results marked in bold.
Obs, observed; SIR, standardized incidence ratio; CI, confidence interval
